# Account of Diseases in an Eastern District of London, from the 20th of July, to the 20th of August, 1801

**Published:** 1801-09

**Authors:** 


*Account of Diseases in an Eastern District of London, from the 20th of July, to the 20th of August,* 1801.

| | No. of Cases. | | No. of Cases. |
|---|---|---|---|
| ACUTE DISEASES. | | Cephalalgia | 5 |
| Typhus | 28 | Paralysis | 3 |
| Hepatitis | 1 | Vomitus | 3 |
| Acute Rheumatism | 1 | Diarrhœa | 8 |
| CHRONIC DISEASES. | | Dyspepsia | 8 |
| Tussis | 10 | Chlorosis | 4 |
| Tussis et Dyspnœa | 7 | Menorrhagia | 4 |
| Pleurodyne | 3 | Asthenia | 4 |
| Phthisis Pulmonalis | 2 | Scrophula | 3 |
| Hydrothorax | 2 | Hæmorrhois | 2 |
| Anasarca | 3 | Hypochondriasis | 3 |
| Ascites | 1 | Psora | 4 |

| | |
|---|---|
| Herpes - - - - - - - | 7 |
| Lepra - - - - - - - - | 1 |
| Rheumatifmus Chronicus | 13 |

PUERPERAL DISEASES.

| | |
|---|---|
| Peritonitis - - - - - - | 1 |
| Maftodynia - - - - - - | 2 |
| Menorrhagia Lochialis - | 3 |

INFANTILE DISEASES.

| | |
|---|---|
| Vermes - - - - - - - - | [illegible] |
| Herpes - - - - - - - - | 5 |
| Diarrhœa - - - - - - - | 4 |

Whilft we are under the neceffity of again adverting to a fubject, which we have long been obliged to confider, — the prevalence of the typhoid contagion — we are, at the fame time, happy to announce, that the fymptoms of the difeafe are much lefs formidable than they have heretofore been, and that a fatal termination takes place much lefs frequently. The number of patients affected by it has indeed been rather increafed, and it continues to propagate itfelf very extenfively; but thofe affections of the brain, which formerly conftituted fo prominent a feature of the difeafe, occur lefs frequently, and appear under a milder form.

The fymptoms of Angina alfo, which in the laft Report were mentioned as accompanying the difeafe, have difappeared. The difeafe therefore now appears under a form in which we have heretofore been accuftomed to view it.

Complaints of the ftomach and bowels have been very frequent. Diarrhœas have in feveral inftances been troublefome, and have continued for a confiderable time; but they have not been attended with any unpleafant confequences, unlefs when they have been injudicioufly checked by aftringents; or when, under the idea of fupporting the ftrength of the patient during the increafed evacuation, recourfe has been had to fome warm cordial. Upon a premature check to this evacuation we often hear of pains in the ftomach and bowels, of which the patient did not before complain, and the removal of which is beft accomplifhed by inviting the return of that difcharge which Nature had inftituted with a view to relieve the conftitution.

